# Salivary vitamin D levels among OSCC and normal Indian patients

**DOI:** 10.6026/97320630018884

**Published:** 2022-10-31

**Authors:** Sandra Sagar, Pratibha Raman, S Gheena, R Abilasha, Reshma Poothakulath Krishnan, J Selvaraj

**Affiliations:** 1 Saveetha Dental College and Hospitals, Saveetha Institute of Medical and Technical Sciences, Chennai 600077, India

**Keywords:** OSCC, Vitamin D, Cancer

## Abstract

Oral Squamous Cell Carcinoma comprises nearly 90% of all Oral cancers. Recent studies on Oral Squamous Cell Carcinoma are done with a focus on identification of factors that affect the outcome of treatment, one of which is vitamin D levels. The aim of this study was to evaluate vitamin D3 levels in patients with Oral Squamous Cell Carcinoma and Normal Population with saliva as a biomarker. The study sample comprised of 40 subjects, of whom 20 were patients diagnosed with OSCC and 20 healthy subjects. The salivary samples obtained were assessed using Vitamin D3 ELISA kit. The mean salivary Vitamin D3 levels were 39.09 ng/dl among OSCC patients and 44.49 ng/dl among healthy subjects. Salivary Vitamin D levels in OSCC patients were found to be significantly less compared to the healthy controls. More than 90% of cancers of the oral cavity are Oral Squamous Cell Carcinoma (OSCC). Recent studies are done with a focus on identification of factors affecting the treatment of cancer, one of which is vitamin D levels. The aim of the study was to evaluate salivary vitamin D3 levels in patients with Oral Squamous Cell Carcinoma and in Normal Population. 40 patients were selected for the study. Salivary samples collected were assessed using Vitamin D3 ELISA Kit-EDI Total 25-OH Vitamin D EIA Kit to detect the salivary vitamin D levels. Mean Salivary Vitamin D3 levels were found to be 39.09 ng/dl in OSCC patients and 44.49 ng/dl in healthy subjects. A salivary Vitamin D level in OSCC patients was found to be significantly less compared to the healthy controls. Decreased Vitamin D3 levels in patients with OSCC indicate that Vitamin D3 deficiency increases the rate of cell proliferation, invasion, angiogenesis & metastasis in patients with OSCC. Vitamin D level is likely to reduce the severity of the disease and hence Vitamin D supplementation for OSCC patients in the early stages of treatment and after treatment can be done to enhance the prognosis.

## Background:

Oral cancer is one of the most common of all cancers. Oral cancers unless detected early have a very low survival rate of less than 5 years. Nearly all Cancers that originate in the oral cavity are Oral Squamous Cell Carcinomas (OSCC) [1]. Etiology of Oral cancer is multifactorial. Oral cancer as seen in many cases is due to the exposure of mucosal surfaces to carcinogens for eg: tobacco & alcohol. In some populations betel and areca nut chewing predispose to cancer. Dietary deficiency like Vitamin D can also be contributory to cancer. Immunosuppression due to viral infections like Human Papilloma Virus (HPV), Epstein Barr Virus (EBV), Human Immunosuppressive Virus (HIV) and Hepatitis C Virus (HCV) are also predisposing factors that lead Oral mucosa to malignant changes. Radiation (UV and radiotherapy) may bring about DNA changes leading to Cancer. Ill-fitting dentures, irritation of sharp tooth over a prolonged period of time can cause cancer. Thus a host of extrinsic and intrinsic factors bring about changes in cellular and molecular levels which lead to malignancy [1, 2]. However Oral cancers can also occur without any such predisposing factors. Recent evidence show that Oncogenes which result from activation of proto-oncogenes and tumour suppressor genes which when inactivated can also cause cancer in some syndromes [3]. Early detection therefore is very important during diagnosis. One of the reasons which hinder early diagnosis is patient compliance because of the invasive techniques used like fine needle aspiration, biopsy etc., and imaging techniques like CT and MRI scan which are expensive. Saliva is a potential biomarker which can be obtained easily and assessed, and is non-invasive. It has been shown that active form of Vitamin D which is 1, 25-dihydroxyvitamin D not only regulates calcium homeostasis and bone mineralisation but also has shown antitumor activities. It exerts growth inhibitory effects by induction of apoptosis, cell cycle arrest and differentiation in cancer cells. It has been demonstrated in animal studies that 1,25D3 inhibits growth of SCC by apoptosis. Treatment options that promote apoptosis will help in management of OSCC [4]. Therefore, it is of interest to evaluate Salivary Vitamin D3 levels in normal healthy patients with those diagnosed as Squamous cell Carcinoma in order to evaluate the role of vitamin D3 in prevention and treatment outcome of OSCC.

## Materials and methods:

The present study was approved by the Institutional Human Ethical Committee of Saveetha Dental College. The patients were thoroughly informed about the study. The informed consents were signed by the patients before being included in the study. During the study, no therapeutic interventions were made and the patient's data were kept confidential. No costs were inflicted on patients for carrying out laboratory tests. The sample size was calculated (n=20) in each group at α=0.05 and a study power of 80% using G power software version 3.1. The criteria of patient's selection to be included in the study were patients of age group 25 to 70 years old. In the case group, 20 patients histopathologically diagnosed as Oral Squamous Cell Carcinoma were included. In the control group,20 normal healthy subjects with no systemic disorders were included. The exclusion criteria were patients taking any Vitamins D supplementation, patients undergoing chemotherapy, radiotherapy or any surgery(except biopsy), systemic diseases affecting the levels of salivary vitamin D levels such as parathyroid, rickets, osteomalacia and sarcoidosis. Patients with any active infections such as hepatitis, HIV, tuberculosis, chronic kidney disease, liver disease were excluded. Malnourished patients and patients with known metastasis were also excluded from the study. In the present study, a total of 40 patients were selected (20 patients with OSCC and 20 healthy subjects). After taking medical histories, clinical examinations and completing the required checklists, and after the individuals signed informed consent forms, a 2-ml salivary sample was collected from each subject. Vitamin D total (25-hydroxy vitamin D) kit was used with the Enzyme Linked Immuno Sorbent Assay (ELISA) method to determine and compare salivary and serum levels of vitamin D between the healthy individuals and those with OSCC.

### Statistical analysis:

Data were analysed with paired-t-test, using SPSS version 23.0. A p value of less than 0.05 was considered to be statistically significant.

## Results:

In order to compare salivary levels of vitamin D in two groups, paired t-test was used. The results of t-test showed that there is a significant difference between the levels of salivary vitamin D in healthy subjects (mean 44.49ng/dl) and salivary vitamin D levels in people with squamous cell carcinoma (mean 39.09 ng/dl) (p-value = 0.010). The results clearly indicate that the level of salivary vitamin D in patients with OSCC was comparatively less than that of healthy subjects (Table 1 - see PDF) (Figures 1 to 3 - see PDF).

## Discussion:

Oral Squamous cell carcinoma unless detected early has a survival rate as low as 5 years. Early detection thus will help reduce the mortality and morbidity of OSCC. The etiology of OSCC in many cases begins with the exposure of mucosal surfaces to topical carcinogens of which alcohol and tobacco plays a major part [5]. However OSCC can also occur without any of these predominant factors. Not all of these cancers have a 'precancerous state'. The etiology of OSCC is multifactorial and could be due to either intrinsic or extrinsic factors. The role of Nutritional and dietary factors in the development of OSCC has been well documented in previous studies. The prevalence of Vitamin D deficiency among OSCC patients raises the question of this deficiency in the pathogenesis of cancer [1]. Lack of early detection of OSCC especially with those without a presenting symptom or a 'precancerous' state could be because of the diagnosing methods used. Biopsy and fine needle aspiration are invasive procedures which require good patient compliance. Imaging systems like the CT scan and MRI besides being expensive lack clear understanding for criteria for diagnostic use. Bio-markers can be classified as genomic, proteomic, or metabolomic. Genomic biomarkers that are helpful in estimating the risk of regional and hematogenous dissemination of malignant oral squamous cells are Integrin α3 and integrin β4. Genomic biomarkers that are used to predict radio-resistance in OSCC tissue are vascular endothelial growth factor, B-cell lymphoma-2, claudin 4, yes-associated protein 1 and MET proto-oncogene, and receptor tyrosine kinase. Cluster of differentiation factor 34 is a salivary biomarker that can identify recurrence potential of oral squamous cell carcinoma (OSCC) [2-4]. The active form of Vitamin D (1alpha, 25-dihydroxycholecalciferol) prevents tumour cell proliferation through its apoptotic actions as shown by numerous studies. Vitamin D also inhibits cell cycle and growth factors signalling pathways [5]. Thus Vitamin D with its apoptotic properties can be used in the management of OSCC. Previous studies have used serum level of Vitamin D as a useful biomarker and predictor for Cancer. Other studies reported in the literature have shown that OSCC has a receptor for Vitamin D known as the VDR (Vitamin D receptor) [6, 7, 8 & 9]. Osafi et al. in an in vitro study suggested activation of two apoptotic pathways (caspase and bcl:bax) by Vitamin D as responsible for its anti-OSCC effects [8]. The importance of Vitamin D in Cancer prevention has been investigated previously and has shown that it reduced toxicities in patients with advanced cancer [10, 11]. The present study estimated the levels of Salivary Vitamin D levels in OSCC patients and then compared them with salivary levels of healthy subjects of South Indian Population in an attempt to find any association between Vitamin D and OSCC. The study also attempted to find whether Saliva can be used as a reliable bio-marker. Results of this study showed that salivary Vitamin D levels were significantly (p<0.05) lower in OSCC patients when compared with the healthy subjects. The results are consistent with the previous salivary study done by Ayla Bahramian et al. on Iranian population. This study is also consistent with previous serum studies done on the Middle Eastern population [11, 12, 13& 14]. The severe Vitamin D deficiency in patients with OSCC observed in the study was earlier reported by other investigators [12, 13]. Studies done by Grimm et al. showed a 100% deficiency and study done by Anand et al. showed a 76.4% deficiency. Adisa et al. did a study on black African subpopulation and found an increase in the expression of vitamin D receptor protein (VDR) in patients with oral SCC and concluded that lowering vitamin D levels and, consequently, increasing VDR, could have a potential effect on the development of SCC in these patient [15, 16, 17 & 18]. Results from this study show that deficiency of 5ng/dl in OSCC patients when compared with the normal patients. This raises the question of whether Vitamin D deficiency could play a role in the etiology of OSCC. In a study done by Grimm et al. the first evidence of VDR (Vitamin D receptor) expression was seen in OSCC patients and concluded that apoptosis induction of VDR+ cells in OSCC by natural or synthetic Vitamin D compounds could be useful in chemoprevention. Further studies are required to know whether supplementation of Vitamin D during the early stages of OSCC could change the outcome of treatment [14, 19, 20]. Early detection of OSCC will play a crucial role in the management. The present study has shown that Saliva can be used as a reliable biomarker. It's non-invasiveness and ease of access along with clinical correlation will certainly help in early diagnosis.

## Conclusions:

The result of the present study clearly establishes that saliva can be used as a reliable bio-marker for assessing Vitamin D deficiency. Its non-invasiveness and ease of access make it a better option as a diagnostic tool. The present study clearly indicates that supplementation of Vitamin D is likely to reduce the severity of Oral Squamous Cell Carcinoma. So assessment of Vitamin D levels in patients attending Clinics should be made a routine investigative procedure. The study also indicates that Management of OSCC patients with supplementation of Vitamin D during early stages and after treatment will certainly change the outcome of the treatment.

## References

[1] Vokes EE (1993). N Engl J Med.

[2] Silverman SJr, Gorsky M (1990). J Am Dent Assoc.

[3] Santosh ABR (2016). J Cancer Res Ther..

[5] Sagar S, Mogit Gupta Y (2020). Drug Invention Today.

[6] Ogbureke KUE (2011). Oral Cancer Tech Inc.

[7] Jou YJ (2010). Analytica Chimica Acta.

[8] https://publications.iarc.fr/.

[9] Sargeran K, Murtoma H (2006). The Journal of Craniofacial Surgery.

[11] Yingyu Ma (2013). Cancer.

[13] Grimm M (2013). Targeted Oncology.

[14] Grimm M (2015). Medicina Oral, Patología Oral Cirugía Bucal.

[15] Osafi J (2014). Journal of Dietary Supplements.

[18] Russel DS (2010). Journal of Comparative Pathology.

[19] Anand A (2017). Contemporary Oncology.

[20] Bochen F (2018). OncoImmunology.

